# Exploring the Discourse Around Zyn Nicotine Pouches on Instagram and TikTok: Content Analysis

**DOI:** 10.2196/88825

**Published:** 2026-07-23

**Authors:** Arpita Tripathi, Beth Hoffman, Christine Larkin, Piper Narendorf, Chaim Kittredge, Coltin Kunz, Jaime E Sidani

**Affiliations:** 1 University of Pittsburgh, 130 De Soto Street, Pittsburgh, PA, United States, 1 6099376163; 2 Rutgers Institute for Nicotine and Tobacco Studies, Rutgers University, , New Brunswick, NJ, United States; 3 VISIMO Inc, Pittsburgh, PA, United States

**Keywords:** oral nicotine pouches, social media, Zyn, nicotine, nicotine and tobacco product

## Abstract

**Background:**

Oral nicotine pouches, such as Zyn, have rapidly grown in popularity in the United States, with sales increasing from 83.2 million cans in 2020 to 385 million cans in 2023. This growth has occurred alongside concerns about youth use. At the same time, Zyn’s visibility on social media has also expanded, where youth-targeted content may shape perceptions and influence product uptake.

**Objective:**

This content analysis of Instagram and TikTok Zyn-related posts aimed to (1) examine their sentiment and content, (2) assess youth appeal, (3) identify potential misinformation, and (4) report the most frequently used hashtags in the selected posts as indicators of platform-specific framing and audience targeting.

**Methods:**

In March 2024, we collected 10,502 Instagram posts and 609 TikTok posts with #Zyn. We used a systematically developed codebook to guide the analysis of a random Instagram subsample (n=1200) and all TikTok posts (n=609). Interrater reliability was assessed using a Cohen κ of more than 0.80 and percent agreement.

**Results:**

Many coded posts expressed positive sentiment (n=789/887, 88.9%) and normalized Zyn use through comedic content (n=308/887, 35.2%). Posts revealed youth-targeted themes, including appealing flavors (n=417/887, 87.6%); Zyn usage methods (n=159/887, 18.1%); and associations with sports, athletic, and gym settings (n=70/887, 8%). Both platforms contained posts with potential misinformation. While TikTok featured more influencer-generated content, Instagram showcased more business and commercial content.

**Conclusions:**

Findings suggest that Zyn-related social media content may appeal to youth. Zyn and other oral nicotine pouches are often presented favorably with engaging content, underscoring the need for updated regulatory strategies to address potential misinformation and their appeal to youth.

## Introduction

Oral nicotine pouches (ONPs) are prefilled, tobacco-free, flavored or nonflavored, dissolvable pouches containing nicotine salts that can be used sublingually [1]. They are one of the least expensive nicotine delivery products and are cheaper than flavored e-cigarettes and cigarettes [2]. Recently, public health experts have raised concerns about the potential health effects of ONPs, considering the known associations between nicotine and neurological and cardiovascular disorders [3], as well as the impact of nicotine pouches on oral health [4]. ONPs may also contain a higher dose of nicotine—an addictive substance—compared to other tobacco-free products and nicotine replacement therapy products [1]. As ONPs are relatively new, nonindustry funded research on their chemical composition and health effects is limited. A recent scoping review found that most studies on nicotine content, pharmacokinetics, and toxicity were industry-funded [1]. One of the few independent studies analyzed 37 ONPs, revealing wide variations in total and free nicotine levels. It suggested that increased pH in some products could lead to blood nicotine levels comparable to snuff or snus [1]. Another recent study found significant dependence among adult ONP users and noted that most users reported at least one adverse event, with mouth lesions and upset stomach being the most frequent [5].

ONPs are the only category of nicotine products for which youth use has not recently declined in the United States [6]. Results from the 2024 National Youth Tobacco Survey found that approximately 1.8% of middle and high school students were current users of ONPs, an increase from 1.5% the previous year [6]. Preliminary analysis of the 2025 National Youth Tobacco Survey indicates that past-30-day ONP use was 1.7% among middle- and high-school students, which is approximately the same as in 2024 [7]. Another study, conducted in 2021, found that 6.4% of respondents aged 13‐20 years reported current use of ONPs, and 11.1% reported ever using them [8]. Similarly, ONP use is rising globally, with approximately 5% of youth reporting ever using them in Canada and New Zealand, and 10% of youth reporting ever using them in England in 2024 [9].

Of current adolescent ONP users in the United States, 24.5% reported using Zyn, which was the most popular oral nicotine product [10]. Zyn—owned by Swedish Match, a subsidiary of the tobacco firm Philip Morris International—has the highest market share in the United States, with over 58.5% of all ONP unit sales in 2022 [11]. Zyn’s sales increased from 83.2 million cans in 2020 to 385 million cans in 2023, indicating a consistent surge in popularity [12]. The product’s popularity in the United States led Philip Morris International to acknowledge a shortage in June 2024. The following month, the company announced a US $600 million investment in a new Zyn production facility [13].

The regulatory status of ONPs is rapidly evolving. In 2022, the US Food and Drug Administration (FDA) announced its regulatory authority over tobacco-free products that contain synthetic nicotine—including ONPs—to ensure that the products are regulated under the same standards as traditional tobacco products [14]. In January 2025, the FDA authorized the marketing of 20 Zyn products [15]. More recently, the FDA launched a pilot program to streamline and increase the efficiency of ONP authorization applications [16], which includes Zyn.

Despite the recent authorizations, Zyn is not yet authorized as a modified-risk tobacco product (MRTP). An MRTP is a nicotine or tobacco product authorized by the FDA to be marketed as a reduced-risk or reduced-harm product when compared to the risks associated with traditional cigarettes. As of 2026, Zyn currently has a pending application with the FDA to be classified as an MRTP, and if granted MRTP approval, it would allow for different risk exposure messaging compared to traditional tobacco products [17,18].

Of particular concern are Zyn’s marketing descriptors, including the usage of terms such as “flavor ban approved,” “smooth,” “chill,” and “tobacco-free” [19,20], since such terms can attract new users and promote continued use [21]. Furthermore, 44.4% of all unique ONP advertisements were sponsored by Zyn, with “freedom” as the most common theme (30.7%) [17]. The perception of Zyn as being tobacco-free, as well as its various flavor options, may add to its appeal among youth and young adults; a 2021 survey of US young adults found that 17% of respondents incorrectly believed that ONPs contain neither tobacco nor nicotine [22].

The growing online presence of Zyn’s marketing is concerning. A 2024 Ad Watch report indicated that Zyn’s digital marketing spending surged from US $2 million in 2022 to US $28 million in 2023 [23]. Campaigns included banner advertisements on the Entertainment and Sports Programming Network, Google mobile apps, and news sites like The Washington Post. Although Zyn’s official Facebook and Instagram pages claim no influencer partnerships, “unbranded” pro-Zyn content, often driven by youth culture, has amassed significant views on platforms like Instagram and TikTok [23]. Research also suggests that exposure to nicotine and tobacco product (NTP) content on video-based platforms, including Instagram and TikTok, has been associated with NTP initiation and a positive attitude toward NTPs [24].

Considering that 46% of adolescents are online almost constantly, and Instagram and TikTok are 2 of the most popular platforms used by youth [25], it is important to categorize and analyze Zyn and other ONP-related content to which they may be exposed in order to inform regulatory efforts to protect consumers [26]. Relatedly, recent literature has examined Zyn-related content on social media platforms, including TikTok and Instagram, and identified themes related to the normalization of Zyn use [26], primarily among male individuals [23,26,27]; the portrayal of Zyn as a relatively harmless product [23,26,27]; and its presence in digital pop culture [27,28]. While past studies have conducted content analyses focused on marketing, demographics, health effects, and product types, there has been little examination of misinformation, post sentiment, or the strategies individuals use, such as the use of hashtags, to share Zyn-related posts across social media platforms. Addressing these gaps is important because platform-specific content may shape youth and young adults’ perceptions of ONPs [29] or contribute to the spread of misleading or unsupported claims [30]. Thus, this content analysis of both Instagram and TikTok Zyn-related posts aimed to (1) examine their sentiment and content, (2) assess youth appeal, (3) identify potential misinformation, and (4) report the most frequently used hashtags in the selected posts to characterize platform-specific content framing [31].

## Methods

### Data Collection

All posts from Instagram and TikTok with the hashtag Zyn (ie, #Zyn) were collected on March 3, 2024. The collected posts were originally published between January 29, 2012, and February 3, 2024, on Instagram, and between March 7, 2019, and February 3, 2024, on TikTok. We used #Zyn because Zyn was the primary focus of this study and is the leading ONP brand in the United States. However, we recognize that this single-hashtag strategy may have excluded relevant posts using other nicotine pouch hashtags, slang terms, alternative spellings, brand comparisons, or no hashtag.

TikTok data (609 posts) was gathered using the open-source package “*TiktokPy*,” which retrieves popular posts based on TikTok’s proprietary algorithm [32]. Instagram data (10,502 posts) was collected via Apify, an online data collection platform that uses its own data collection algorithm [33]. In both cases, the collected data represented content that was publicly available through #Zyn, including profile names and URLs, post content, captions, and engagement metrics such as likes. All additional hashtags used in the posts were extracted for analysis.

The sampling frames in the study differed by platforms. Apify’s Instagram hashtag scraper is designed to retrieve public Instagram posts associated with one or more hashtags. In contrast, *TikTokPy* is a Python scraping package (Python Software Foundation) that extracts TikTok data without application programming interface keys. This implies that retrieved posts may reflect TikTok’s platform-specific ranking, availability, and algorithmic display at the time of collection. Therefore, the Instagram and TikTok datasets should be interpreted as platform-specific samples of publicly available #Zyn content. These platform-specific differences are an important challenge for social media data collection and reproducibility [34]. Similar data collection approaches have been used in prior public health studies, including studies using NTP data [28,35].

### Codebook Development and Coding Procedure

The codebook was developed using a hybrid deductive-inductive coding approach [36]. The lead authors developed an initial codebook for human coding based on prior NTP research [37-39] and a review of 25 Instagram and 25 TikTok posts from the original dataset. To validate the first draft of the codebook, the initial draft was used to code a random subsample of 100 posts (50 from Instagram and 50 from TikTok). All posts in the initial subsample were double coded by trained human coders. To refine the codebook, all disagreements were resolved through an iterative adjudication process conducted over 3 rounds. The final codebook was applied to the remaining posts, which were coded by trained human coders.

The 609 TikTok posts were double-coded by 2 independently working coders, and annotations and memos were written to highlight key themes identified within each post. Any differences in coding were adjudicated by the coders during weekly sessions with the lead author.

For Instagram, considering the large volume of Instagram posts and because manual coding is resource intensive, a random subsample of 1200 posts was selected to be coded by 4 coders. This approach has been recommended for qualitative analysis of large datasets of social media data [40]. The approach also helped balance the feasibility of this study while retaining a large number of posts for content analysis. During coder training and reliability assessment, Instagram posts were double-coded in sequential batches. During this period, interrater reliability was assessed using the Cohen κ and percentage agreement across main categories, including relevance, account type, content characteristics, post purpose, and potential misinformation (see the “Coding Categories” section for codebook category explanations). Once a Cohen κ of greater than 0.8 was achieved for the main categories mentioned above, the remaining Instagram posts were single-coded. In total, 1809 posts were coded between the 2 platforms.

### Coding Categories

Each post was first coded for availability, indicating whether the post was accessible at the time, and for English language. Relevant posts, defined as those that focused on Zyn or ONPs, were categorized by account type, post purpose, post content, sentiment, appeal, and potential misinformation. Account type codes included commercial, influencer (≥10,000 followers) [41], athlete, or youth or young adult-appearing. For posts coded as youth or young adult appearing, coders' self-assessment was used to make the decision. Adolescent-appearing individuals were defined as those individuals who appeared to be between the ages of 11 and 17 years, and young adult-appearing individuals were defined as those individuals who appeared to be between the ages of 18 and 20 years. These codes were based on criteria including self-reported age, school or college references, profile biography, captions, and visual appearance. This assessment, based on observable characteristics in posts or user profiles, has been used in prior studies [42]. Furthermore, account type and appearance codes were not mutually exclusive. For example, a post authored by an influencer could also be coded as youth- or young adult–appearing if the individual featured in the post appeared to fall within those age categories.

Posts were also classified by their primary purpose: informational, comedic, or product-focused, with youth-appealing content identified. The purpose category was mutually exclusive. When a post appeared to serve more than 1 purpose, such as being both comedic and product-focused, coders assigned the category that best reflected the post’s primary purpose. A post coded as informational included posts that discussed statistics, prevalence, and health effects, among other data, related to the use of Zyn or ONPs. A post coded as product-focused referred to those that emphasized showcasing, displaying, or promoting Zyn or ONPs. All posts that could not be categorized under any of the above categories were coded as “others.”

Posts were coded for overall themes, including depicting or suggesting Zyn or ONP use, and sentiment, including pro-Zyn or pro-ONP, anti-Zyn or anti-ONP, or neutral toward these products. Content codes included sports or gym depictions, flavors, personal experiences, and product promotion.

Potential misinformation was flagged for unverifiable claims. Potential misinformation was defined as any explicit or factual claim about ONPs, nicotine, health effects, cessation, NTP safety, and so on, that was either contradicted or could not be verified in the peer-reviewed literature as of November 2024. It was coded in 2 stages. First, the coders applied a broad interpretation of potential misinformation. Later, these flagged posts by the coders were reviewed by senior researchers (redacted for review). Medline and Google Scholar were used to assess claims against peer-reviewed literature; content contradicting or lacking scientific support (as of November 2024) was deemed misinformation. Content codes were not mutually exclusive unless specified otherwise. See Table 1 for the overarching themes used in the codebook and Table 2 for the complete codebook.

**Table 1.** Definitions of main themes and their descriptions.

Overarching theme	Description
Relevant	Identifies whether Zyn or oral nicotine pouches was a main topic of the post and whether the post met inclusion criteria for analysis.
Account type	Captures characteristics of the account or individuals featured in the post, including whether the account was commercial, athlete-associated, influencer-associated, or featured adolescents or young adults.
Presence of NTPs^a^	Identifies whether the post displayed or referred to Zyn use, Zyn product imagery, alcohol, or other NTPs.
Sentiment	Captures the overall attitude toward Zyn expressed in the post, including positive or negative sentiment.
Content	Describes the substantive content of the post, including personal experiences, flavor references, athletics, bodybuilding or gym-related content, and unrelated content that used Zyn imagery.
Purpose	Captures the primary purpose or function of the post, such as informing viewers, entertaining through humor, or displaying or discussing the product.
Appeal	Identifies whether the post contained elements that appeared to appeal to specific population groups, including youth and racial, ethnic, sexual, or gender minority groups.
Misinformation and misleading health claims	Captures posts containing potentially false, unsupported, or misleading factual claims about Zyn, nicotine, health effects, safety, or product effects.

a NTP: nicotine and tobacco product.

**Table 2.** Definitions for all codebook themes and their frequencies.

Content category and code	Definition	Example	TikTok (n=453), n (%)	Instagram (n=434), n (%)
Account type				
Commercial	The post is authored by a supplier, distributor, or marketer of Zyn or ONP^a^ products.	A local distributor shows their Zyn stock in the post.	84 (18.5)	207 (47.7)
Athlete	The post is authored by or features a school, college, or professional athlete.	A college athlete discusses their use of Zyn before big games.	5 (1.1)	4 (0.9)
Adolescent appearance	The post features any individual who appears to be between the ages of 11 and 18 years.	A boy who appears to be middle-school age uses Zyn in the video.	12 (2.7)	0 (0)
Young adult appearance	The post features any individual who appears to be between the ages of 18 and 20 years.	A man who appears to be college age uses Zyn in the video.	110 (24.2)	24 (5.5)
Influencer	The post is authored by an account with 10,000 or more followers.	An individual with 16,000 followers makes a post featuring Zyn.	128 (28.3)	58 (13.4)
Presence of nicotine and alcohol products				
Zyn or ONP use	The post displays or suggests the use of Zyn or ONPs.	The post shows an individual placing or keeping Zyn in their mouth.	155 (34.1)	4 (0.9)
Zyn or ONP image	The post displays a Zyn or ONP package, can, pod, or something resembling the product.	The post shows an individual holding a Zyn pack with the product taking up most of the screen.	27 (5.6)	262 (60.4)
Alcohol	The post displays or refers to alcoholic beverages.	The post contains an advertisement for margarita-flavored Zyn.	43 (9.5)	15 (3.5)
Other nicotine and tobacco products	The post displays or refers to another nicotine or tobacco product.	The post displays a pack of cigarettes or nicotine vape alongside Zyn.	23 (5.1)	23 (5.3)
Sentiment				
Pro-Zyn or pro-ONP	Positive sentiment toward Zyn or ONPs is expressed in the post.	A student mentions that using Zyn helps them focus on their work.	409 (90.3)	380 (87.6)
Anti-Zyn or anti-ONP	Negative sentiment toward Zyn or ONPs is expressed in the post.	An adult recommends against Zyn use in the post.	13 (2.9)	4 (0.9)
Content				
Personal or proximal experience	The post contains or describes an individual’s personal or proximal experience with Zyn or ONPs.	An athlete reports feeling dizzy and lightheaded the first time they used Zyn.	34 (7.5)	11 (2.5)
Flavorings	The post shows or mentions a flavor of Zyn or ONPs.	A post advertises a Zyn pack that is visibly cool mint flavor.	118 (26.0)	299 (68.9)
Athletics	The post contains content related to sports or athletic activity.	A post references or depicts tennis, basketball, soccer, running, swimming, or similar sports.	31 (6.8)	25 (5.8)
Bodybuilding or gym	The post contains content related to gym use, bodybuilding, muscle gain, or fitness routines.	An individual displays a bodybuilding workout and states that Zyn is part of their routine.	11 (2.4)	3 (0.7)
Unrelated content with Zyn	The post uses Zyn or ONP product imagery to promote or discuss unrelated content.	A retailer of nonnicotine products replaces the text on a Zyn tin to advertise their own product.	8 (1.8)	37 (8.5)
Purpose				
Informational	The post provides information about Zyn/ONPs, including statistics, product use, trends, or claims.	A user presents statistics on Zyn use over the past year.	17 (3.7)	19 (4.4)
Comedic	The post uses humor, jokes, memes, or entertainment-based framing related to Zyn or ONPs.	A user makes a joke about Zyn.	185 (40.9)	123 (28.3)
Product-focused	The post displays, discusses, promotes, or centers the Zyn or ONP product.	An individual shows the Zyn product with no further explanation.	35 (7.7)	234 (53.9)
Appeal				
Youth	The post contains elements that may appeal to children or adolescents.	A post displays a Zyn flavor that resembles the flavor of well-known candies.	116 (25.6)	231 (53.2)
Black	The post contains elements that appear to appeal to Black individuals or communities.	A post uses images or themes related to Black identity or empowerment.	0 (0)	0 (0)
Hispanic	The post contains elements that appear to appeal to Hispanic individuals or communities.	A post uses Spanish language or Hispanic cultural imagery.	0 (0)	0 (0)
Sexual or gender minorities	The post contains elements that appear to appeal to sexual or gender minority communities.	A post uses LGBTQ+^b^ signifiers such as a pride flag.	0 (0)	0 (0)
Misinformation and misleading health claims				
Potential misinformation	The post contains factual claims about Zyn or ONPs that are contradicted by or could not be verified in peer-reviewed literature.	A user claims that Zyn use can improve eyesight.	21 (4.6)	24 (5.5)

a ONP: oral nicotine pouch.

b LGBTQ: lesbian, gay, bisexual, transgender/transsexual, queer.

### Analysis

#### Content and Thematic Analysis

Frequencies and percentages were calculated for each code. Following this, the senior authors (redacted for review) independently analyzed a total of 300 posts, consisting of 150 TikTok posts and 150 Instagram posts. For this thematic analysis, each author began by individually reviewing 50 posts (25 from each platform), consulting the coders’ notes, and taking their own detailed notes. The authors (redacted for review) then met to discuss and identify emerging themes. This process was repeated, with each author analyzing an additional 25 posts from each platform before reconvening to refine and expand the identified themes. The review process continued until thematic saturation was achieved. Additionally, due to the high number of posts coded for athletic content, youth-targeting, and potential misinformation, the lead authors (redacted for review) conducted a separate in-depth qualitative analysis of all posts within these categories.

#### Hashtag Analysis

All hashtags used in the captions of the Instagram and TikTok posts coded as relevant were collected and separately processed using R software (version 4.1.2; R Foundation for Statistical Computing). Text-cleaning was performed on all hashtags on both platforms, including converting text to lowercase and removing duplicates. Later, the frequency of each hashtag was calculated on both platforms. To qualitatively compare hashtags, the lead authors (AT, BH, and JS) independently—and later as a group—reviewed the most frequently occurring hashtags across the 2 platforms and compared them for overlap, platform, and broader thematic patterns.

### Ethical Considerations

This study was reviewed by the University of Pittsburgh Human Research Protection Office (STUDY22080079) and was determined not to constitute human participant research because it used publicly available social media data and involved no direct interaction or intervention with individuals. Therefore, informed consent was not required. To protect privacy and confidentiality, no usernames, profile information, or other directly identifying information is reported, and findings are presented in aggregate and/or deidentified form.

## Results

### Content Analysis

Among all coded posts, 453 out of 609 (74.4%) TikTok posts and 434 out of 1200 (36.2%) Instagram posts were coded as relevant to Zyn or ONPs. All subsequent percentages are reported among relevant coded posts unless otherwise stated.

Among relevant coded posts, 27.0% (n=122) of the TikTok posts and 5.5% (n=24) of the Instagram posts appeared to be posted by youth or young adults. While 48.0% (n=207) of the Instagram accounts were coded as commercial, only 18.5% (n=84) of TikTok accounts were coded as commercial. Additionally, 28.0% (n=128) of TikTok posts and 13.0% (n=58) of Instagram posts were made by influencer accounts. The majority of posts—90.3% (n=409) on TikTok and 87.6% (n=380) on Instagram—showcased pro-Zyn or pro-ONP content. The primary purpose of the posts was to show comedic content, with 40.9% (n=185) on TikTok and 28.3% (n=123) on Instagram, or to showcase products related to Zyn or ONPs, with 7.7% (n=35) on TikTok and 53.2% (n=231) on Instagram. Active Zyn or ONP use was more common on TikTok than Instagram, appearing in 34.1% (n=155) of TikTok posts compared with 1.0% (n=4) of Instagram posts. Close-up images of Zyn or ONP cans or pods were more common on Instagram, appearing in 60.3% (n=262) of Instagram posts compared with 5.6% (n=27) of TikTok posts. Alcohol-related content appeared in 9.5% (n=43) of TikTok posts and 3.5% (n=15) of Instagram posts, while other NTPs appeared in 5.1% (n=23) of TikTok posts and 5.3% (n=23) of Instagram posts. No posts on either platform were coded as appealing specifically to Black, Hispanic, or sexual or gender minority audiences. See Table 2 for descriptives of the main coding categories.

### Thematic Analysis

**Figure 1.**
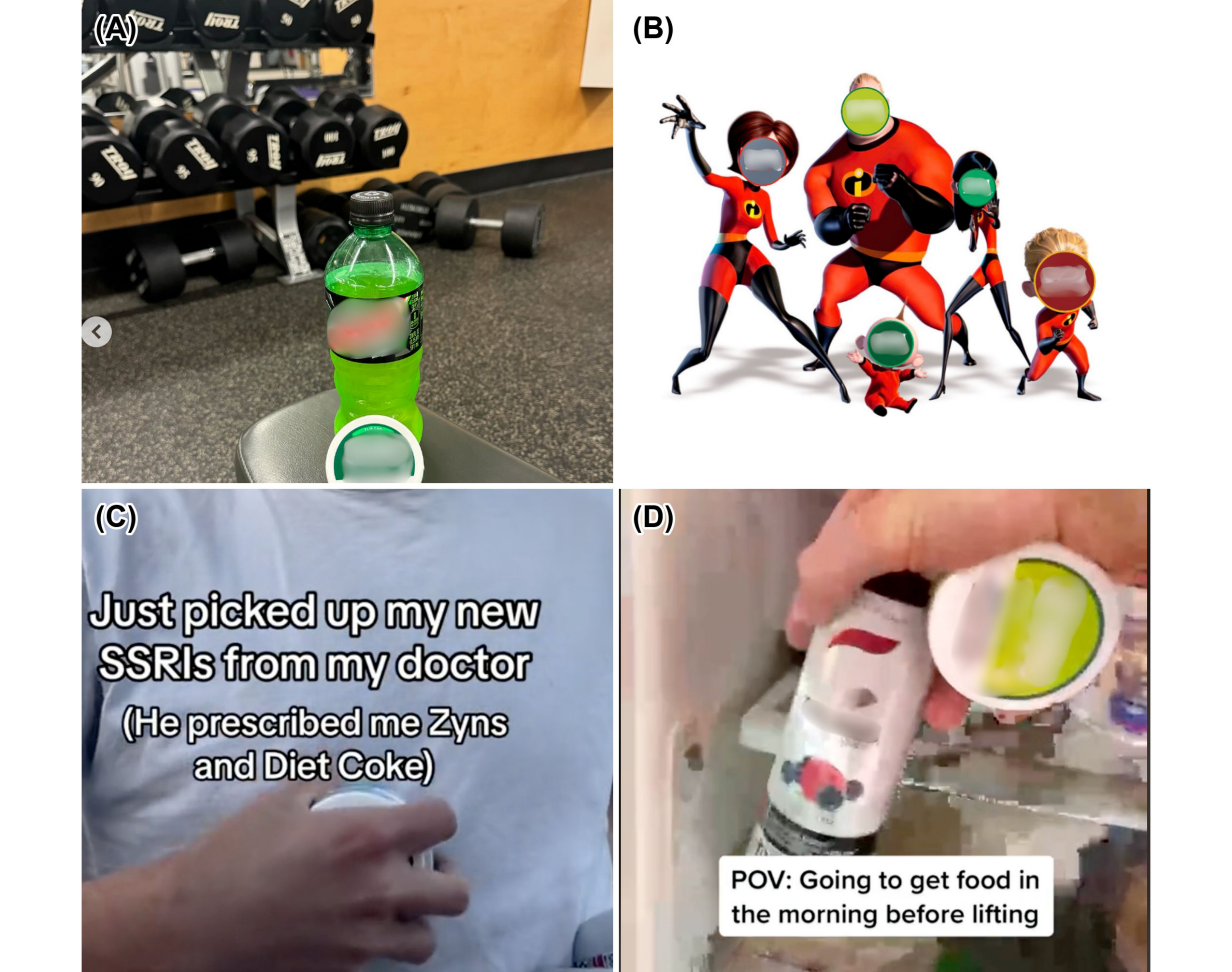
Example images representing each of the main themes explored in the paper. (A) The image highlights the use of Zyn in gym and athletic settings. (B) The image depicts Zyn flavors associated with popular cartoon characters. (C) The image exemplifies misleading health claims, humorously equating Zyn and Diet Coke to selective serotonin reuptake inhibitors (SSRIs), which are commonly prescribed antidepressants. (D) The image portrays Zyn paired with energy drinks, suggesting their use as substitutes for food before workouts. POV: point of view.

Figure 1 presents illustrative examples of each of the themes discussed in this section.

### Misleading Health Claims and Misinformation

Of the 24 out of 434 Instagram posts (5.5%) coded for potential misinformation, and on TikTok, 33 out of 453 posts (7.2%) were coded as potential misinformation. These posts were reviewed by senior authors, and it was found that 9 out of 24 (37.5%) Instagram posts and 21 out of 33 (63.6%) TikTok posts contained misinformation. Common misinformation themes promoted Zyn as a smoking or vaping quit aid or claimed unproven health benefits, such as cognitive enhancement or antidepressant effects. Some anti-Zyn posts also contained unverified claims, such as Zyn causing mouth cancer or enamel decay. Others falsely advertised nonexistent Zyn concentrations (eg, 150 mg).

Thematic analysis revealed that misleading health claims were also common, particularly in athletic and youth-targeted posts. These posts implied benefits such as improved performance or weight loss. One Instagram post showed a Zyn-sponsored hockey team outscoring a Gatorade-sponsored team, suggesting that Zyn enhances athletic performance. A TikTok video depicted a man taking Zyn and immediately doubling his bench press record. Weight loss–related posts often used imagery to imply that Zyn or ONPs could serve as a meal replacement, featuring individuals pairing Zyn or ONPs with coffee for breakfast or prioritizing Zyn or ONP purchases over groceries.

### Youth Appeal

About 53% (n=231) of the posts on Instagram and 26% (n=116) of the posts on TikTok were coded as appealing to youth. On Instagram, these posts featured flavors of Zyn or ONPs (eg, mint, fruit, and coffee). Some posts also displayed images of Zyn with fake flavors inspired by food items such as Cheetos. Other youth-appealing posts included Zyn cans depicted in various art styles and memes using Zyn for comedic effect. On TikTok, most youth-appealing posts prominently featured popular pop-culture movies, television shows, and celebrities. For example, 1 post used popular animated characters to depict a fictional island with Zyns. Furthermore, posts on TikTok merged the term “Zyn” into other cultural references to create attention-grabbing terms such as Zynbawe, Zyndiana Jones, and Zynley Cup. Finally, a recurring youth-appealing theme involved meme-based posts that linked Zyn or ONPs to childhood nostalgia using candy-related imagery. For example, some posts showed a “same feeling—different age” format, displaying colorful candies captioned “same feeling,” followed by Zyn or ONP cans captioned “different age.” These posts suggested that Zyn or ONPs evoked feelings similar to those associated with candies, framing Zyn or ONPs through youth-oriented nostalgic imagery.

### Normalization of Zyn or ONP Use

A prominent theme that emerged across both platforms was the normalization of Zyn use through the depiction of its integration into everyday life activities. On TikTok, 7.5% (n=34) of posts shared everyday experiences with using Zyn or ONPs, including the use of Zyn or ONPs with meals, drinks, and workouts. One video, titled “boy dinner,” featured the use of Zyn alongside beer, ground beef, and sauce, while another showcased Zyn cans as Christmas decorations. On Instagram, 2.5% (n=11) of posts shared everyday experiences with using Zyn or ONPs. For example, commercial accounts reinforced normalization by marketing Zyn-related accessories like can holders, branded t-shirts, and recycling bins. Some posts framed vaping as outdated, using slogans like “Vaping is so 2019, join the revolution.”

### Use of Zyn or ONP for Athletics and Gym

Zyn was frequently linked to sports like golf, hockey, football, and baseball, with golf being the most mentioned. On TikTok, 6.8% (n=31) of the content was related to sports or athletics, while 2.4% (n=11) was specifically related to the gym. For example, in a TikTok video, a golfer denied using Zyn despite visibly having it in their upper lip, subtly normalizing its use. Another video showed a person placing Zyn pouches in hockey players’ mouths mid-game. On Instagram, 5.8% (n=25) of the content was related to sports or athletics, while less than 1% (n=3) was specifically related to the gym. For example, on Instagram, an edited image of a well-known golfer holding a Zyn can promoted the product, and a “Zynley Cup” trophy made from Zyn cans was widely shared. Gym-related posts often framed Zyn as a preworkout product. One Instagram post featured a gym scene with dumbbells, a sugary drink, and a can of Zyn.

### Hashtag Analysis

In addition to the hashtag #Zyn, there were a total of 4247 and 6935 additional hashtags used on TikTok and Instagram, respectively. The most frequently used hashtags on Instagram included niche brand names of other ONPs such as Lyft, Nicopods, Swedish Snus, Whitefox, Skruf, Siberia Snus, and Ace SuperWhite. Conversely, the most frequently used hashtags on TikTok were fyp (“for you page”), fy (“for you”), and viral.

Both platforms used hashtags to describe characteristics of Zyn, such as nicotine pouches, snus, tobacco-free, smokeless, nicotine, Nic, or 6 mg (a term that describes a popular strength of Zyn pouches). Posts on both platforms also used informal terms to describe the action of using Zyn, such as “upper decky,” “lip/gum pillow,” and “upper decky lip cushy.” Furthermore, posts on both platforms frequently used wordplays on the term Zyn to create terms such as ZynTok, ZynGang, Zynbabwe, ZynFam, ZynFlavor, ZynUSA, and Zynfluencer.

Finally, a trend on TikTok was observed where sports-related hashtags, such as golf, hockey, golftiktok, and barstoolsports, were frequently used. Notably, “ferda,” a term popular among hockey players meaning “for the boys,” was also prevalent. Additionally, hashtags reflecting masculine themes or stereotypes, including cheddy, the boys, and frat were identified. Beyond male-specific content, TikTok featured more lifestyle-related hashtags such as collegetok, college life, and beer. See Multimedia Appendix 1 for the full list of hashtags.

## Discussion

### Principal Findings

This content analysis of Instagram and TikTok posts that used the hashtag #Zyn found that most expressed pro-Zyn or pro-ONP sentiment. While few Instagram posts appeared to have been created by young people, approximately one-quarter of TikTok posts were created by them. The use of comedy or entertainment was common in Zyn or ONP-related posts. Specific themes emerged from this analysis, including the depiction of flavors, a connection to athletics and working out, and the normalization of Zyn or ONP use. The analysis of hashtags was consistent with the content and thematic findings.

Influencer marketing, a focus on Zyn or ONP-imagery, and overwhelmingly pro-Zyn or pro-ONP content were important findings of this study. Considering that influencer marketing significantly impacts consumer behavior among younger audiences [43], the prevalence of pro-Zyn or pro-ONP content shared by influencers may contribute to a favorable image of the product, making it appear more appealing and socially acceptable. While this study does not directly examine audience perception or behavior, it may inform future studies aimed at assessing the impact of influencers and Zyn or ONP imagery on product uptake. Furthermore, the integration of promotional content within influencers’ posts can make it difficult for young audiences to recognize the commercial intent, thereby influencing product uptake [44]. Finally, exposure to Zyn imagery may increase the desire to consume ONPs, as indicated by previous research examining behavioral and neural responses to e-cigarette advertising [45].

Several findings from this study may warrant the attention of policymakers. First, although Zyn does not maintain an official presence on TikTok or Instagram [46], this study highlights the potential impact of user-generated content on young people. Over 50% (231/453) of Instagram posts and 26% (116/453) of TikTok posts in this study appealed to youth, with 27% (n=134) posted by young creators. Content often featured flavors, memes, animations, and pop culture trends. Although these findings do not directly measure youth exposure or behavioral effects, they align with research on how visually engaging and culturally relevant content attracts young audiences to novel nicotine products [8]. Likewise, flavors have been a key driver of e-cigarette use among youth [47], highlighting the need to monitor and restrict youth access to flavored Zyn or ONPs. In its authorization of Zyn marketing, the FDA states that it will *impose stringent marketing restrictions for digital, TV, and radio* to reduce youth exposure to marketing, and that the company planned to implement a variety of measures to restrict youth access [15]. However, this study found that much of the Zyn- or ONP-related content on TikTok and Instagram was user-generated, suggesting that it will be available on these platforms despite mitigation efforts. Considering that exposure to e-cigarette content on social media is reaching youth and is associated with increased susceptibility to the use of these products [48], addressing both user and manufacturer-posted content is crucial.

This study found that the normalization of Zyn or ONP use through its integration into everyday life was commonly observed on both platforms. Posts often portrayed Zyn or ONP as a socially acceptable part of daily life, integrating it into meals, beverages, workouts, and holiday celebrations. Commercial accounts further reinforced this with Zyn or ONP-related merchandise. This relatability of content via normalization may enhance its appeal to viewers and, by extension, may influence the uptake and use of ONPs [8,49]. Furthermore, these findings align with prior research that highlights the role of social media in integrating nicotine products within cultural and recreational settings, thereby reducing harm perceptions and increasing their social acceptability among younger audiences [26,50]. Relatedly, the hashtag analysis found frequent use of terms like ZynTok, ZynGang, and Zynbabwe, reflecting Zyn’s popularity and a unique and engaging collective identity for its users. This viral, community-driven culture broadens its reach and further normalizes Zyn use, especially among younger audiences [51].

Misinformation and misleading health claims about Zyn or ONPs were present on both platforms, potentially influencing harm perception. Previous research on e-cigarette content has identified common misinformation themes, including claims of safety, significantly reduced harm compared to cigarettes, unproven nicotine benefits, and exaggerated product risks [37,38]. The Zyn or ONP misinformation themes observed in this study were almost identical, including pro-Zyn misinformation about Zyn or ONPs as a proven quit aid and Zyn or ONPs having unproven health benefits, as well as anti-Zyn or anti-ONP posts about health harms that have yet to be confirmed by research. These findings highlight the need for a comprehensive approach to addressing NTP misinformation rather than focusing on individual products. Additionally, these findings suggest the need for further research examining the presence of misinformation on Instagram and TikTok, as most NTP misinformation research to date has focused on X, formerly known as Twitter.

Moreover, this study extends previous research examining e-cigarette misinformation to include posts that were misleading. In some posts, misinformation was not clearly stated; instead, a combination of text and visual imagery implied that using Zyn or ONPs can provide performance or health benefits, such as improving athletic performance. These findings may offer a springboard for developing machine learning or other automated techniques to categorize misleading NTP posts on visual platforms. Future research can also examine whether viewing misleading posts is associated with susceptibility to Zyn or ONP initiation.

Finally, this study identified key cross-platform differences in Zyn or ONP-related content. The content on TikTok primarily focused on describing the uses and experiences with Zyn or ONPs. This content was diverse and user-generated. In contrast, content on Instagram was mainly driven by commercial accounts that displayed visually attractive images of flavored Zyn or ONPs. Hashtag analysis reinforced these distinctions—Instagram hashtags often included other ONP brands, targeting a brand-focused audience, while TikTok featured broader hashtags like #fyp and #viral, enhancing content discoverability. However, the distribution of hashtags on TikTok was skewed, reflecting generic hashtags such as #fyp being more frequently used than hashtags related to the content of the posts, such as hashtags with sports or fitness. This may indicate that TikTok hashtag practices reflect algorithmic visibility strategies more than the substantive content of the posts. Overall, these findings highlight the need to consider platform-specific dynamics and adopt tailored regulatory strategies.

### Limitations

This study has several limitations. First, the sampling strategy limits representativeness and reproducibility. Data collection was restricted to posts containing the hashtag #Zyn, which may have excluded relevant Zyn or ONP-related content that used other hashtags, alternative spellings, or no hashtag. In addition, data were collected on a single day, making this study a cross-sectional snapshot of publicly available #Zyn content rather than an assessment of trends over time.

Second, Instagram coding was limited to a random subsample of 1200 posts from the 10,502 collected Instagram posts because manual content coding of the full dataset was not feasible. Although the subsample was randomly selected, it was not statistically compared with the full Instagram dataset on engagement metrics, posting patterns, or other observable features. Therefore, Instagram findings should be interpreted as descriptive of the coded random subsample.

Third, this study analyzed social media content rather than audience exposure, perceptions, susceptibility, or product use. As a result, the findings cannot determine whether youth viewed the content, how they interpreted it, or whether exposure influenced attitudes or behavior. The results of the study must, therefore, be interpreted as descriptive findings that may aid in hypothesis generation for future studies.

Fourth, qualitative interpretation and manual coding of social media posts can be inherently subjective. To improve consistency and reproducibility, the team used a structured codebook, trained coders, double coding, reliability assessment, and adjudication procedures that have been used in previous studies [37-39]. However, some constructs, including apparent age, youth appeal, and misinformation, required judgment based on observable post or profile features and available scientific evidence.

Fifth, misinformation classification was limited by the evidence available at the time of the review. Posts were coded as misinformation or misleading health claims only when the claims were contradicted by, unsupported by, or could not be verified in peer-reviewed literature available as of November 2024. Some claims may have since been confirmed, refuted, or clarified by newer evidence.

Finally, only posts that were accessible at the time of coding were analyzed, so posts that became unavailable, were deleted, or were made private before coding might have been missed.

### Public Health Implications and Conclusions

In the evolving NTP landscape, video platforms offer insights into discourse on emerging products like ONPs. This study provides a cross-sectional description of publicly available #Zyn content on TikTok and Instagram and highlights prevalent positive sentiment toward Zyn or ONPs, including content with youth-appealing features. These findings may inform future tobacco control surveillance and policy discussions and reveal parallels in promotional tactics with e-cigarette marketing [52]. However, because this study did not measure youth exposure and product uptake, the findings should be interpreted as descriptive rather than causal. Overall, the study also underscores the importance of online monitoring and suggests the need to strengthen US online advertising regulations to curb youth-targeted marketing. For example, Australia recently introduced a regulation where ONPs are treated as therapeutic and prescription-only products, and advertising them on any platform, including social media, is banned [53]. Furthermore, Canada recently introduced a regulation for emerging nicotine replacement products, including ONPs, that prohibits the use of advertisements, labels, or packaging that can be appealing to youth. Taken together, these examples suggest that regulations on ONP advertising should be considered not only for paid promotions and advertisements but also for influencer-based marketing and youth-appealing imagery that is prevalent on popular social media platforms [54].

## Supplementary material

10.2196/88825 Multimedia Appendix 1 List of hashtags.

## Abbreviations

FDA: US Food and Drug Administration
MRTP: modified-risk tobacco product
NTP: nicotine and tobacco product
ONP: oral nicotine pouch
