# Supplementary material for: Exploring the Discourse Around Zyn Nicotine Pouches on Instagram and TikTok: Content Analysis
Source: JMIR Infodemiology. 2026 Jul 23;6:e88825. doi: 10.2196/88825 (PMC13392921; doi:10.2196/88825)
Supplement: Multimedia Appendix 1 [file infodemiology-v6-e88825-s001.docx]

| **Appendix 1: List of 50 most popular hashtags in posts coded as relevant with #Zyn.** | | | |
| --- | --- | --- | --- |
| **Instagram** | | **TikTok** | |
| Hashtag | Frequency | Hashtag | Frequency |
| nicotine | 139 | fyp | 263 |
| tobaccofree | 138 | snus | 65 |
| snus | 128 | viral | 61 |
| nicotinepouches | 99 | snusmemes | 60 |
| lyft | 97 | zynygang | 53 |
| nicopods | 95 | snusljus | 52 |
| snuff | 89 | nicotinepouches | 51 |
| snuslife | 85 | zyntok | 49 |
| swedishsnus | 85 | snustown | 48 |
| nicotinepouch | 82 | foryou | 45 |
| whitefox | 82 | upperdecky | 45 |
| skruf | 74 | snussking | 39 |
| siberiasnus | 70 | fyp | 38 |
| meme | 67 | zynbabwe | 35 |
| smokeless | 63 | ferda | 32 |
| snusdosa | 63 | foryoupage | 28 |
| acesuperwhite | 62 | virals | 25 |
| chewingtobacco | 56 | fypã‚· | 24 |
| tobacco | 54 | cheddy | 23 |
| zyns | 53 | freezertarps | 23 |
| zynpouches | 52 | zyns | 23 |
| pouches | 50 | golf | 22 |
| nic | 48 | collegetok | 20 |
| freezertarps | 46 | comedy | 17 |
| zynlife | 46 | lippillow | 16 |
| 6mg | 43 | beer | 15 |
| smokefree | 43 | college | 15 |
| upperdecky | 43 | funny | 15 |
| flavor | 42 | hockey | 14 |
| funny | 42 | nicotine | 14 |
| lippillow | 42 | barstoolsports | 13 |
| zynflavor | 42 | capcut | 12 |
| zynner | 42 | freezertarps | 12 |
| 3mg | 41 | snus | 12 |
| dailyzyn | 41 | trending | 12 |
| zynnachino | 41 | foryou | 11 |
| zynnation | 41 | theboys | 11 |
| zynusa | 41 | velo | 11 |
| zynlove | 40 | zynbabwe | 11 |
| zynsanity | 39 | frat | 10 |
| gumpillow | 36 | fraternity | 10 |
| vape | 36 | meme | 10 |
| zynfam | 36 | zynfluencer | 10 |
| velo | 34 | meme | 9 |
| chewbags | 33 | tuckercarlson | 9 |
| odens | 33 | upperdeckylipcushies | 9 |
| nordicspirit | 31 | collegelife | 8 |
| sale | 31 | drinking | 8 |
| f4follow | 29 | drunkdriving | 8 |
| l4likes | 29 | golftiktok | 8 |
